# The Efficacy and Safety of Procedural Sedoanalgesia with Midazolam and Ketamine in Pediatric Hematology

**DOI:** 10.4274/tjh.2014.0149

**Published:** 2015-12-03

**Authors:** Sema Aylan Gelen, Nazan Sarper, Uğur Demirsoy, Emine Zengin, Esma Çakmak

**Affiliations:** 1 Kocaeli University Faculty of Medicine Hospital, Department of Pediatrics, Division of Pediatric Hematology, Kocaeli, Turkey

**Keywords:** Sedoanalgesia, Ketamine, Midazolam, Invasive procedure

## Abstract

**Objective:**

The aim of this study is to investigate the efficacy and safety of sedoanalgesia performed outside the operating room by pediatricians trained in advanced airway management and life support.

**Materials and Methods:**

Midazolam and ketamine were administered consecutively by intravenous route under cardiorespiratory monitoring for painful procedures of pediatric hematology.

**Results:**

A total of 115 patients had 237 sedoanalgesia sessions. Sedation time was 24.02±23.37 s and sedation success was 92.5% (Ramsay scores of ≥5). Patient satisfaction was high. The recovery time was 28.81±14.4 min. Although statistically significant (p<0.01) increases in systolic and diastolic blood pressure, heart rate, and respiratory rate were observed without clinical importance, they improved without any intervention. No severe adverse events were observed.

**Conclusion:**

Sedoanalgesia with intravenous midazolam and ketamine for pediatric hematology and oncology patients’ painful minor invasive procedures performed in an optimally equipped setting outside the operating room by pediatricians trained and certificated in advanced airway management and life support is effective and safe.

## Abstract (tr)

**Amaç:**

Çalışmamızın amacı, ileri yaşam desteği konusunda eğitimli pediatristler tarafından ameliyathane dışında gerçekleştirilen ağrılı işlemlerde uygulanan sedoanaljezinin etkinliği ve güvenilirliğinin araştırılmasıdır.

**Gereç ve Yöntemler:**

Pediatrik hematolojinin ağrılı işlemlerinde, kalp-solunum monitorizasyonu yapılarak midazolam ve ketamin ardarda intravenöz yolla uygulanmıştır.

**Bulgular:**

Yüz on beş hastaya 237 sedoanaljezi uygulandı. Sedasyon süresi 24,02±23,37 saniye, sedasyon başarısı %92,5 idi (Ramsay skoru ≥5). Hasta memnuniyeti yüksekti. Uyanma süresi 28,81±14,45 dakika idi. İşlemler sırasında sistolik ve diastolik tansiyon artışı, kalp hızı ve solunum sayısında artış istatistiksel olarak anlamlı (p<0,01) bulunmasına rağmen, klinik olarak anlamlı değildi ve herhangi bir girişim yapılmadan düzeldi. Ciddi yan etki gözlenmedi.

**Sonuç:**

Pediatrik hematoloji ve onkoloji hastalarının ağrılı minör invazif işlemlerinde, intravenöz midazolam ve ketamin ile sedoanaljezi; uygun donanıma, monitorizasyona sahip bir ortamda ve ileri yaşam desteği konusunda eğitimli ve sertifikalı pediatristler tarafından gereğinde yapıldığında, ameliyathane dışında da etkin ve güvenilirdir.

## INTRODUCTION

Lumbar puncture, bone marrow aspiration/biopsy, and intrathecal therapy are painful procedures. In patients with leukemia, traumatic lumbar puncture due to poor patient stabilization is a diagnostic dilemma and may cause seeding of the blasts into the cerebrospinal fluid from circulation [1,2]. The burden of the procedure under inadequate sedoanalgesia can lead to refusal of the diagnostic procedure or treatment [3].

In this study, the aim was to evaluate the efficacy and safety of procedural sedoanalgesia performed by pediatricians and hematologists trained in advanced airway management and life support.

## MATERIALS AND METHODS

This prospective study was planned by pediatric hematologists. The ethics committee of the center approved the study and written informed consent was obtained. Physicians were trained in advanced life support and had a proficient command of the characteristics and pharmacology of the sedatives/analgesics. One of the physicians performed the invasive procedure, and the other administered the drugs, assisted in patient monitoring, and recorded the vital signs and sedation and recovery times. During the lumbar punctures and intrathecal therapy a nurse assisted in proper positioning of the patient.

Sedation time (the period to induce sedation after the administration of both drugs) and recovery time (the period until the patient was awake with age-appropriate behavior and age-appropriate oriented responses to verbal and motor stimuli after the procedure was accomplished) were recorded.

Sedation was initiated with midazolam (0.1 mg/kg/dose by slow intravenous infusion, maximum 10 mg) and continued with ketamine (1 mg/kg/dose by slow intravenous bolus, maximum 100 mg). Level of sedation was assessed according to the modified Ramsay scale (Table 1). When the score was 5 or 6, which was considered as satisfactory sedation, the procedure was initiated. A score of below 5 was rated as unsatisfactory sedation. Patients were followed by the study nurse for 4 h for any adverse events. Severe adverse events were defined as cardiovascular collapse, airway and respiratory events including hypoxemia requiring resuscitation, and allergic reactions.

### Statistical Analysis

Statistical analysis was performed using SPSS 2.0 (SPSS Inc., Chicago, IL, USA). For evaluation of demographic characteristics descriptive statistics were used, and for intergroup comparison of the parameters that had normal distribution the paired samples t-test was used.

## RESULTS

Between May 2012 and May 2013, a total of 237 invasive procedures (bone marrow biopsy/aspiration, intrathecal chemotherapy) were performed in 115 children (9.4±4.5 years, range: 10 months to 19.5 years) with sedoanalgesia.

Median sedation time was 24.02±23.37 s (range: 1-300 s). Median recovery time was 28.81±14.45 min (range: 5-90 min). In 87% (n=207) of the sessions no additional dose was administered. Due to prolongation of the procedures or unsatisfactory sedation 1 additional dose of midazolam, 1 additional dose of ketamine, and 2 additional doses of ketamine were administered in 2 (0.8%), 29 (12.2%), and 1 (0.8%) of the sedoanalgesia sessions, respectively. Out of 32 additional doses, 17 (53%) were administered due to multiple painful procedures in the same session.

Oxygen saturation was over 90% in all the patients during sedation and at recovery. There was no apnea, respiratory depression, or need for assisted ventilation/intubation. None of the patients required flumazenil administration. No severe adverse events were observed. Vital signs are shown in Table 2. A significant increase in systolic and diastolic blood pressure, heart rate, and respiratory rate was observed during sedation and when the procedure was completed compared to baseline values (p<0.01). However, these increases were not clinically significant, and after recovery, they returned to reference values (p<0.01). There was hypersalivation during sedation, when the procedure was completed, and when awake in 16.9%, 24.5%, and 5.1% of the sessions, respectively (p<0.01). Simply wiping the secretions was enough; no aspiration was required. After the procedures in 6.8% (n=16), 5.1% (n=12), 1.7% (n=4), and 1.3% (n=3) of the sessions, hallucinations, vomiting, agitation, and pain at the procedure site were respectively recorded, but patients recovered without any therapeutic intervention. Hallucinations were seen during recovery but they were transient and self-limited. The overall adverse event rate was 14.8% (n=35). Sedation was successful in 92.5% (n=219) of the procedures. All the procedures were completed successfully and all the outpatients could be discharged on the same day. Patient satisfaction was high; when painful procedures were repeated all the patients and/or caregivers preferred the same sedoanalgesia.

## DISCUSSION

In developing countries during painful procedures many centers perform no sedoanalgesia due to limited numbers of anesthesiologists, busy operation rooms, and inadequate training in sedoanalgesia, advanced airway management, and life support [4]. In many studies, it has been shown that sedation and analgesia during painful procedures were administered with equally good results by pediatricians who had received advanced life support training [5,6,7,8].

When midazolam and ketamine are used alone, respiratory depression with midazolam and dysphoric reactions (irritability, depression, etc.) with ketamine may occur. When midazolam is used with ketamine, faster analgesia, amnesia, and fewer side effects occur [9,10,11]. Oxygen desaturation may increase with addition of high-dose midazolam [12]. Therefore, additional doses of ketamine are preferred. In some previous studies with midazolam and ketamine the incidence of oxygen desaturation was between 4.8% and 12%, whereas Ozdemir et al. reported no oxygen desaturation [7,13,14,15,16]. In the present study none of the patients’ oxygen saturation dropped below 90%. Compared to propofol, the combination of ketamine and midazolam was associated with less hypoxemia [13,14].

In recent reports, similar to our findings, a significant increase in cardiovascular parameters was seen due to ketamine’s sympathomimetic action via inhibition of catecholamine reuptake, but these parameters returned to baseline values at recovery and no treatment was required [2,14,15,16,17].

In other published studies the sedation time was similar to that of the present study [2,14,16]. Parker et al. showed that more than 70% of the patients woke up in 30 min, similar to our result [9]. Short sedation and recovery time seems a good feature of the drug combination.

Overall adverse event rate in our study was comparable to those of some other studies [18,19,20]. Ketamine may cause airway obstruction, laryngospasm, and aspiration by increasing tracheal and bronchial secretions. Agents such as atropine and glycopyrrolate can be used to reduce the increased secretions [15,21,22]. The hypersalivation rate was higher compared to those in the literature; this may be due to no atropine or glycopyrrolate administration. Prone position during and after the procedure prevented obstruction of the airway and aspiration was not required.

Venipuncture is another painful procedure for outpatient children, but oral, nasal, and rectal administrations of midazolam can provide slower sedation and this may cause a delay in the procedure. The intramuscular route is also painful and may require additional injections. With the intravenous route, sedoanalgesia can be achieved faster and, if necessary, additional doses and drugs for cardiopulmonary resuscitation can be administered easily.

## CONCLUSION

With adherence to the published guidelines, sedoanalgesia with intravenous midazolam and ketamine performed by two physicians, trained in airway management and life support, in an optimally equipped setting outside the operating room is safe and efficient. Sedoanalgesia reduces the physical and psychological trauma of the invasive procedures for the patients, parents, and physicians and increases the success of the procedures.

## Figures and Tables

**Table 1:**
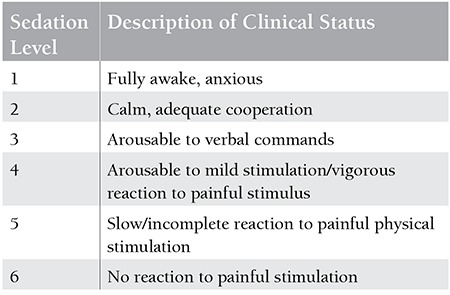
Sedation score (modified Ramsay score).

**Table 2:**
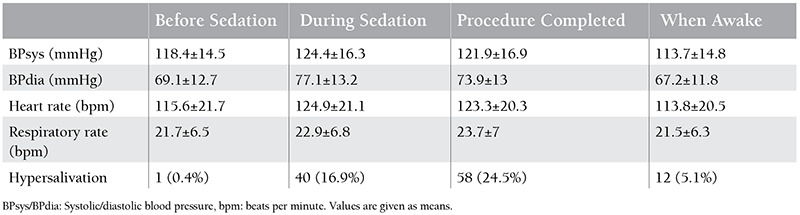
Cardiovascular parameters, respiratory rate, and hypersalivation in invasive procedures under midazolam/ketamine sedoanalgesia.

## References

[1] Maurizi P, Russo I, Rizzo D, Chiaretti A, Coccia P, Attina G, Ruggiero A, Riccardi R (2014). Safe lumbar puncture under analgo-sedation in children with acute lymphoblastic leukemia. Int J Clin Oncol.

[2] Meyer S, Aliani S, Graf N, Reinhard H, Gottschling S (2003). Sedation with midazolam and ketamine for invasive procedures in children with malignancies and hematological disorders: a prospective study with reference to the sympathomimetic properties of ketamine. Pediatr Hematol Oncol.

[3] Sitaresmi MN, Mostert S, Schook RM, Veerman AJ (2010). Treatment refusal and abandonment in childhood acute lymphoblastic leukemia in Indonesia: an analysis of causes and consequences. Psychooncology.

[4] Iannalfi A, Bernini G, Caprilli S, Lippi A, Tucci F, Messeri A (2005). Painful procedures in children with cancer: comparison of moderate sedation and general anesthesia for lumbar puncture and bone marrow aspiration. Pediatr Blood Cancer.

[5] American Society of Anesthesiologists Task Force on Sedation and Analgesia by Non-Anesthesiologists (2002). Practice guidelines for sedation and analgesia by non-anesthesiologists. Anesthesiology.

[6] Pitetti RD, Singh S, Pierce MC (2003). Safe and efficacious use of procedural sedation and analgesia by nonanesthesiologists in a pediatric emergency department. Arch Pediatr Adolesc Med.

[7] Borker A, Ambulkar I, Gopal R, Advani SH (2006). Safe and efficacious use of procedural sedation and analgesia by non-anesthesiologists in a pediatric hematology-oncology unit. Indian Pediatr.

[8] Monroe KK, Beach M, Reindel R, Badwan L, Couloures KG, Hertzog JH, Cravero JP (2013). Analysis of procedural sedation provided by pediatricians. Pediatr Int.

[9] Parker RI, Mahan RA, Guigliano D, Parker MM (1997). Efficacy and safety of intravenous midazolam and ketamine as sedation for therapeutic and diagnostic procedures in children. Pediatrics.

[10] Pellier I, Mongrial JP, Le Moine P, Rod B, Rialland X, Granry JC (1999). Use of intravenous ketamine-midazolam association for pain procedures in children with cancer: a prospective study. Paediatr Anaesth.

[11] Marx CM, Stein J, Tyler MK, Nieder ML, Shurin SB, Blumer JL (1997). Ketamine-midazolam versus meperidine-midazolam for painful procedures in pediatric oncology patients. J Clin Oncol.

[12] Cheuk DK, Wong WH, Ma E, Lee TL, Ha SY, Lau YL, Chan GC (2005). Use of midazolam and ketamine as sedation for children undergoing minor operative procedures. Support Care Cancer.

[13] Godoy ML, Pino AP, Córdova LG, Carrasco OJA, Castillo MA (2013). Sedation and analgesia in children undergoing invasive procedures. Arch Argent Pediatr.

[14] Gottschling S, Meyer S, Krenn T, Reinhard H, Lothschuetz D, Nunold H, Graf N (2005). Propofol versus midazolam/ketamine for procedural sedation in pediatric oncology. J Pediatr Hematol Oncol.

[15] Karapinar B, Yilmaz D, Demirağ K, Kantar M (2006). Sedation with intravenous ketamine and midazolam for painful procedures in children. Pediatr Int.

[16] Ozdemir D, Kayserili E, Arslanoglu S, Gulez P, Vergin C (2004). Ketamine and midazolam for invasive procedures in children with malignancy: a comparison of routes of intravenous, oral and rectal administration. J Trop Pediatr.

[17] Roback MG, Wathen JE, Bajaj L, Bothner JP (2005). Adverse events associated with procedural sedation and analgesia in pediatric emergency department: a comparison of common parenteral drugs. Acad Emerg Med.

[18] Ozkan A, Okur M, Kaya M, Kaya E, Kucuk A, Erbas M, Kutlucan L, Sahan L (2013). Sedoanalgesia in pediatric daily surgery. Int J Clin Exp Med.

[19] Migdady MI, Hayajneh WA, Abdelhadi R, Gilger MA (2011). Ketamine and midazolam sedation for pediatric gastrointestinal endoscopy in the Arab world. World J Gastroenterol.

[20] Wood M (2013). The use of intravenous midazolam and ketamine in pediatric dental sedation. SAAD Dig.

[21] Wathen JE, Roback MG, Mackenzie T, Bothner JP (2000). Does midazolam alter the clinical effects of intravenous ketamine sedation in children? A double-blind, randomized, controlled, emergency department trial. Ann Emerg Med.

[22] Ramaiah R, Bhananker S (2011). Pediatric procedural sedation and analgesia outside the operating room: anticipating, avoiding and managing complications. Expert Rev Neurother.

